# Identifying Methylation Signatures and Rules for COVID-19 With Machine Learning Methods

**DOI:** 10.3389/fmolb.2022.908080

**Published:** 2022-05-10

**Authors:** Zhandong Li, Zi Mei, Shijian Ding, Lei Chen, Hao Li, Kaiyan Feng, Tao Huang, Yu-Dong Cai

**Affiliations:** ^1^ College of Biological and Food Engineering, Jilin Engineering Normal University, Changchun, China; ^2^ Shanghai Institute of Nutrition and Health, Chinese Academy of Sciences, Shanghai, China; ^3^ School of Life Sciences, Shanghai University, Shanghai, China; ^4^ College of Information Engineering, Shanghai Maritime University, Shanghai, China; ^5^ Department of Computer Science, Guangdong AIB Polytechnic College, Guangzhou, China; ^6^ Bio-Med Big Data Center, CAS Key Laboratory of Computational Biology, Shanghai Institute of Nutrition and Health, University of Chinese Academy of Sciences, Chinese Academy of Sciences, Shanghai, China; ^7^ CAS Key Laboratory of Tissue Microenvironment and Tumor, Shanghai Institute of Nutrition and Health, University of Chinese Academy of Sciences, Chinese Academy of Sciences, Shanghai, China

**Keywords:** methylation, COVID-19, feature selection, decision tree, rule

## Abstract

The occurrence of coronavirus disease 2019 (COVID-19) has become a serious challenge to global public health. Definitive and effective treatments for COVID-19 are still lacking, and targeted antiviral drugs are not available. In addition, viruses can regulate host innate immunity and antiviral processes through the epigenome to promote viral self-replication and disease progression. In this study, we first analyzed the methylation dataset of COVID-19 using the Monte Carlo feature selection method to obtain a feature list. This feature list was subjected to the incremental feature selection method combined with a decision tree algorithm to extract key biomarkers, build effective classification models and classification rules that can remarkably distinguish patients with or without COVID-19. EPSTI1, NACAP1, SHROOM3, C19ORF35, and MX1 as the essential features play important roles in the infection and immune response to novel coronavirus. The six significant rules extracted from the optimal classifier quantitatively explained the expression pattern of COVID-19. Therefore, these findings validated that our method can distinguish COVID-19 at the methylation level and provide guidance for the diagnosis and treatment of COVID-19.

## Introduction

Coronavirus disease 2019 (COVID-19) was announced as a “public health emergency of international concern” by the World Health Organization (WHO) on January 30, 2020 and was assessed as a global pandemic on March 11, 2020 (Rodríguez-Morales et al., 2020; Eurosurveillance Editorial Team, 2020). The causative agent of COVID-19 is a new type of coronavirus, whose complete gene sequence is approximately 79.5% similar to that of severe respiratory syndrome coronavirus SARS-CoV. Therefore, it was named SARS-CoV-2 by the International Virus Laboratory Classification (Zhou et al., 2020; Zhu et al., 2020). SARS-CoV-2 is a group 2B *ß*-coronavirus, which is a linear single-stranded positive-stranded RNA virus. It is similar to other coronaviruses and consists of four structural proteins, namely, spike protein, envelope protein, membrane protein/matrix protein, and nucleocapsid protein. COVID-19 has a huge impact on global public health. According to WHO, SARS-CoV-2 had caused 156,496,592 infections and 3,264,143 deaths worldwide until May 8, 2021, and a total of 1,171,658,745 vaccine doses had been administered worldwide till May 5, 2021 (World Health Organization, 2020). However, definite and effective treatments for COVID-19 are still lacking, and no antiviral drug has been confirmed by a rigorous “randomized, double-blind, placebo-controlled” study.

As early as 1975, researchers (Holliday and Pugh, 1975; Riggs, 1975) found that in vertebrates, cytosine methylation at the CpG site can be used as a genetic marker and can be passed on to the next generation by cell division. In plants and mammals, methylation on the 5th carbon atom of cytosine residues is the most widely studied epigenetic modification. In mammals, cytosine methylation mostly exists on the CG sequence; plants have CHG and CHH methylation (H = A, C, or T) in addition to CG methylation. DNA methylation is relatively stable and can exist persistently during DNA replication. The position of DNA methylation can be determined and its relationship with gene regulation can be explored with the development of whole-genome methylation sequencing technology. The DNA methylation of the promoter region can suppress gene expression by preventing transcription factor accessibility. Moreover, the DNA methylation of the gene body can affect chromatin structure, alternative splicing, and transcription efficiency (Lorincz et al., 2004). In mammals, such as *Homo sapiens* and *Mus musculus*, DNA methylation is necessary to maintain normal embryonic development (Yin et al., 2012; Guo et al., 2014), and abnormal methylation has remarkable effects on diseases (Robertson, 2005). In addition, methylation plays an important role in the regulation of the expression of tissue-specific genes or developmental stage-dependent genes (Gehring and Henikoff, 2007).

Strong evidence showed that epigenetic markers, including histone modifications, DNA methylation, chromatin remodeling, and non-coding RNAs, affect gene expression profiles and increase individual vulnerability to virus infections (Fang et al., 2012; Menachery et al., 2014). Meanwhile, viruses have developed a complex, highly evolved, and coordinated process that can regulate the host’s epigenome, control the host’s innate immune and antiviral defense processes, and thus promote the powerful replication of the virus and the onset of disease (Schäfer and Baric, 2017). Circulating blood DNA methylation profiles are altered in patients with severe diseases, including severe sepsis and pediatric critical illness (Binnie et al., 2020; Güiza et al., 2020).

In this study, we obtained methylation data from 106 SARS-CoV-2-positive patients and 26 SARS-CoV-2-negative patients. Machine learning algorithms, such as Monte Carlo feature selection (MCFS) (Dramiński et al., 2007) and decision tree (DT) (Safavian and Landgrebe, 1991), were applied to identify methylation features and decision rules that clearly distinguish different cases and to build classification models with excellent performance to provide insight into the diagnosis, susceptibility, and potential pathogenesis of COVID-19.

## Materials and Methods

### Datasets

We downloaded the methylation data of the 128 samples from Gene Expression Omnibus with accession number GSE174818 (Balnis et al., 2021), which contains 102 samples from patients with COVID-19 and 26 samples from patients without COVID-19. For each sample, 86,5807 methylation sites were identified by the Illumina Human Methylation EPIC platform.

### Monte Carlo Feature Selection

For the investigated methylation data, features (methylation sites) were much more than sample numbers. Evidently, not all features were related to COVID-19. It is necessary to analyze all features and extract essential ones. As different feature selection methods may produce quite different results, selection of proper methods was quite essential. To our knowledge, MCFS is good at dealing with data containing few samples and large features. Thus, it was adopted in this study.

The MCFS method (Dramiński et al., 2007) is an effective and broadly adopted feature selection method that is composed of various DTs and builds various bootstrap sets with subsets of randomly selected features. First, *m* bootstrap sets and *t* feature subsets are created from the primary data set. Then, one tree is constructed for m  bootstrap sets and t feature subsets. Overall, m × t  DTs are created. The relative importance (RI) score for each feature can be calculated based on the resultant DTs. RI score is calculated as the frequency of a target feature in the growing DT as follows:

$$RI_{f}=\sum_{t=1}^{mt}{\left(w\mathrm{Acc}\right)}^{u}IG\left(n_{f}\left(\tau \right)\right){\left(\frac{\text{no}.\text{in}\;n_{f}\left(\tau \right)}{\text{no}.\text{in }\;\tau }\right)}^{v},$$ (1)

where *f* indicates a feature; wAcc means the weighted accuracy of the DT τ ; $IG\left(n_{f}\left(\tau \right)\right)$ refers to the information gain of node $n_{f}\left(\tau \right)$ ; $\text{no}.\text{in}\;n_{f}\left(\tau \right)$ and no.in τ denote the number of samples of $n_{f}\left(\pi \right)$ and τ , respectively; and u and v are weighting factors, which are set to 1. More significant features have higher RI values. Therefore, the features were sorted in decreasing order based on their RI values in the new feature list after MCFS processing.

The MCFS program used in this study was loaded from http://www.ipipan.eu/staff/m.draminski/mcfs.html. For convenience, the program was run using the default parameters, and *u* and *v* were set to 1.

### Incremental Feature Selection

Although the MCFS method can rank features by their importance, it cannot determine which features are essential. Therefore, the incremental feature selection (IFS) method (Liu and Setiono, 1998) was used to determine the optimal number of essential features required for the classification algorithm. First, IFS yields a series of feature subsets based on step size from the list of features received from the MCFS method described above. For example, when the step size is 5, the first feature subset includes the top 5 features, the second feature subset includes the top 10 features, and so on. Afterward, IFS trains the classifier on the training samples, which contain these features on each feature subset. The best subset was determined based on the evaluation metrics of the obtained model by evaluating this classifier through 10-fold cross-validation (Kohavi, 1995; Chen et al., 2021; Liu et al., 2021; Li X. et al., 2022; Tang and Chen, 2022; Yang and Chen, 2022).

### Decision Tree

DT is one of the most classic machine learning algorithms (Safavian and Landgrebe, 1991). Although it is not very powerful, even much weaker than several strong machine learning algorithms, it also has its merits. In fact, DT is a white-box model, meaning it is possible for users to understand its classification principle. This cannot be achieved for all black-box models, which is always more powerful than DT. In the field of biomedical research, such merit is quite helpful as investigators want to not only build efficient models but also obtain helpful clues to understand the complicated underlying mechanism. Accordingly, DT is widely accepted in the field of biomedical research (Zhang et al., 2021a; Zhang et al., 2021b; Huang et al., 2021; Li Z. et al., 2022; Chen et al., 2022; Ding et al., 2022). Generally, DT uses the IF–THEN format to accomplish classification or regression tasks through a tree structure. It often yields satisfactory performance at a low computational cost. In this work, we applied the Scikit-learn module in Python to build the DT classifier.

### Synthetic Minority Oversampling Technique

As described in the *Datasets Section*, a considerable variation in the sample sizes of patients with COVID-19 was observed. In this case, a classifier with excellent performance is difficult to build, because the predicted results are suitable for the type with the largest sample size. Synthetic minority oversampling technique (SMOTE) (Chawla et al., 2002) was performed in the present work to address this problem. This method ensures that the number of samples in the minority class is equal to the number of samples in the majority class after processing by adding new samples to the minority class. In detail, *x* is a randomly selected sample in the minor class, and some samples with the same class that are closest to *x* are identified. Next, sample *y* is randomly selected from the closest samples mentioned above, and a novel sample is produced by choosing a randomly selected point between *x* and *y* in the feature space. The newly produced sample is deeply associated with *x* and *y*; thus, it has a high probability of belonging to the same class as *x* and *y* and is therefore considered to be in the same class. The above procedure executes several times until the minority class has same number of samples in the majority class.

In this study, we employed the SMOTE procedure acquired from https://github.com/scikit-learn-contrib/imbalanced-learn and directly used the default parameters.

### Performance Measurement

For the binary model used in this study, its predicted results can be counted as a confusion matrix, which contains four entries: true positive (TP), false positive (FP), true negative (TN) and false negative (FN). According to these entries, several measurements can be calculated. In this study, we adopted the following measurements: sensitivity (SN, also called recall), specificity (SP), prediction accuracy (ACC), Matthews correlation coefficient (MCC), precision and *F*_1_-measure (Sasaki, 2007; Powers, 2011; Zhao et al., 2018; Wu and Chen, 2022). They can be computed by

$$\mathrm{SN}=\;\frac{\mathrm{TP}}{\text{TP}+\text{FN}}\text{,}$$ (2)

$$\mathrm{SP}=\;\frac{\mathrm{TN}}{\text{TN}+\text{FP}}\text{,}$$ (3)

$$\mathrm{ACC}=\;\frac{\mathrm{TP}+\mathrm{TN}}{\text{TP}+\text{FN}+\text{TN}+\text{FP}},$$ (4)

$$\mathrm{MCC}=\;\frac{\mathrm{TP}\times \mathrm{TN}-\mathrm{FP}\times \mathrm{FN}}{\sqrt{\left(\mathrm{TN}+\mathrm{FN}\right)\left(\mathrm{TN}+\mathrm{FP}\right)\left(\mathrm{TP}+\mathrm{FN}\right)\left(\mathrm{TP}+\mathrm{FP}\right)}},$$ (5)

$$\mathrm{Precision}=\;\frac{\mathrm{TP}}{\text{TP}+\text{FP}},$$ (6)

$$F_{1}-\mathrm{measure}=\frac{2\times \text{precision}\times \text{recall}}{\text{precision}+\text{recall}}.$$ (7)

Among above measurements, we selected F1-measure as the key measurement as it can better reflect the stability of the model. A higher F1-measure indicates a more robust classification model.

## Results

**FIGURE 1:**
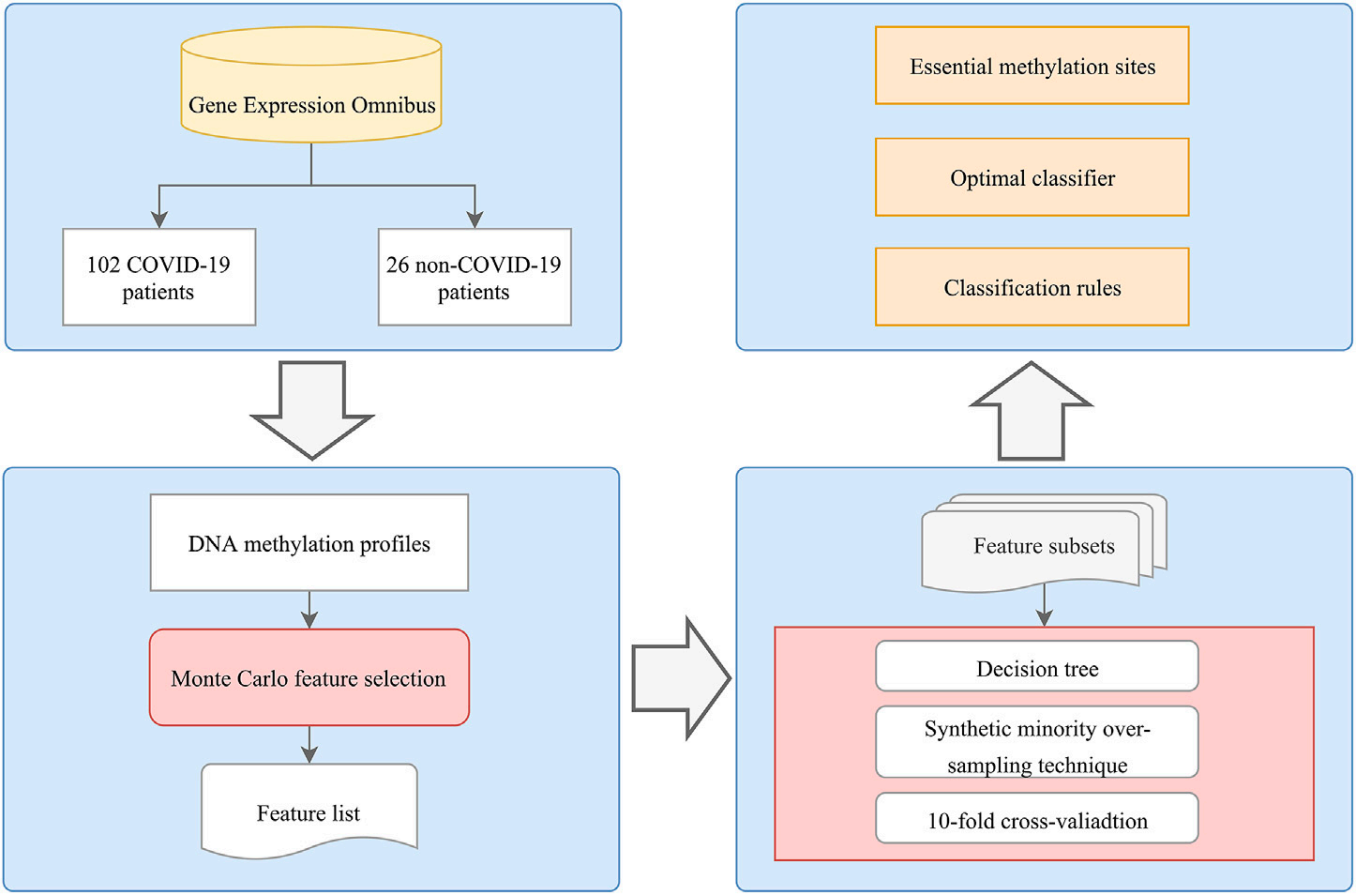
Flowchart of the computational method in this study. A systematic analysis process that integrates feature selection, DT algorithms, and rule learning was applied to identify COVID-19 methylation site features. The optimal classifier, methylation sites, and rules were determined based on the performance of the DT model and the importance of the features in each model.

As shown in Figure 1, we applied an analysis flow to extract key features and build the classification model and rules. The results are summarized in the following sections.

### Results of MCFS Method on Methylation Profiles

We employed the MCFS method to assess the importance of each feature and select key sites from the COVID-19 methylation dataset. These features were ranked in decreasing order of RI scores, and the results are presented in Supplementary Table S1.

### Results of IFS Method With DT

**FIGURE 2:**
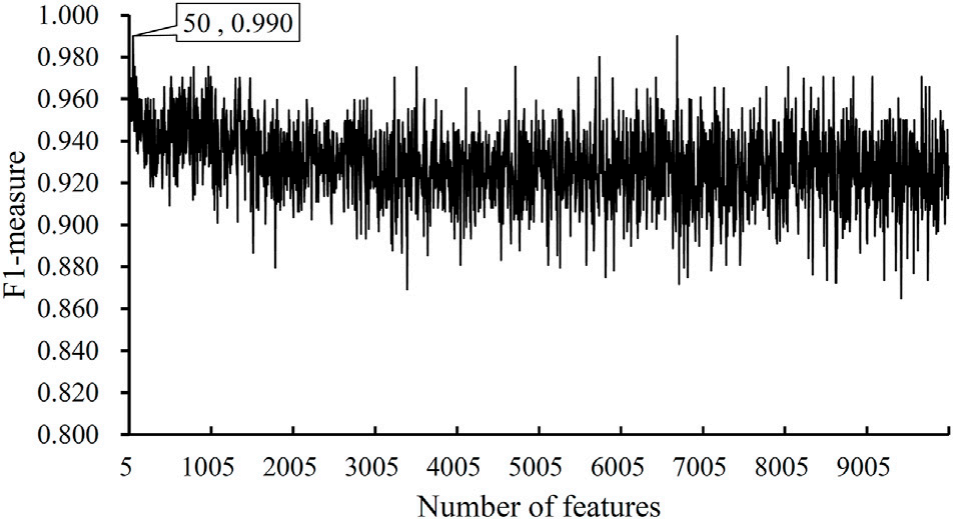
IFS curves obtained by DT classification models on the top 1000 features of the COVID-19 dataset. The model produced the highest F1-measure of 0.990 when the top 50 features were used.

**FIGURE 3:**
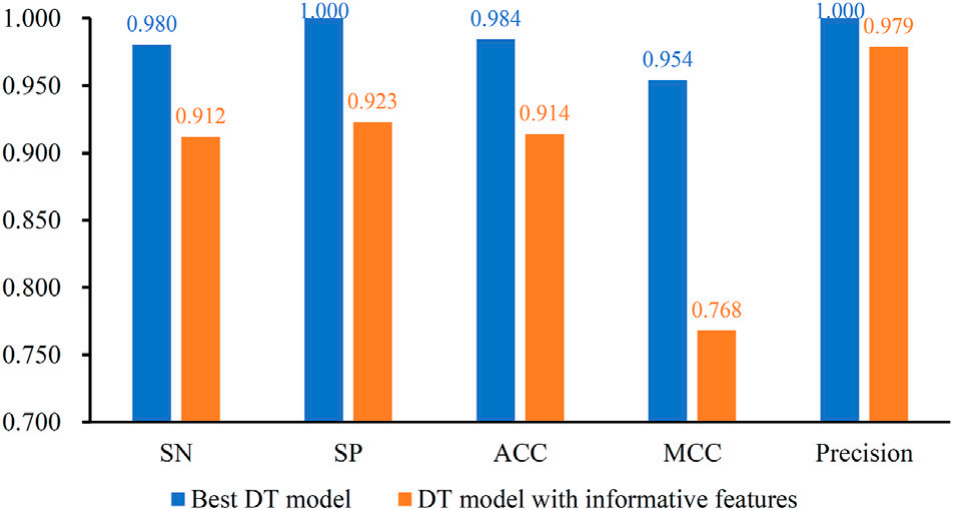
Performance of the best DT model and DT model with informative features. The best DT model is superior to the DT model with informative features.

After the MCFS analysis, we brought the obtained feature list into the IFS with the DT algorithm. The step size of the IFS was set to 5. Since the list was very large, it would take lots of time to consider all possible feature subsets. Furthermore, not all methylation features are related to COVID-19. Thus, only top 10,000 methylation features in the list were considered, that is, 2000 feature subsets were investigated. DT model was constructed on each feature subset and was evaluated by 10-fold cross-validation. The obtained evaluation metrics, including SN, SP, ACC, MCC, precision and F1-measure are listed in Supplementary Table S2. To display the DT models on different feature subsets, we plotted an IFS curve using the number of features as the *X*-axis and F1-measure as the *Y*-axis, which is shown in Figure 2. It can be observed that the highest F1-measure was 0.990, which was obtained by using top 50 features in the list. The other five measurements of such model are illustrated in Figure 3. The ACC and MCC were 0.984 and 0.954, respectively. As for SN, SP and precision, they were 0.980, 1.000 and 1.000, respectively. This result indicated that the constructed optimal DT model has a near-perfect performance and proved the effectiveness of the analysis method.

### Comparison of DT Models With Informative Features

In this study, the IFS method was adopted to extract best features for DT and the best DT model was constructed with these features. In fact, MCFS can yield essential features, called informative features, by only analyzing the methylation data. With these informative features, a DT model can also be built. It is interesting to compare the performance of these two models.

For the methylation data, 257 informative features were obtained by MCFS. The DT model with these features was evaluated by 10-fold cross-validation. The F1-measure was 0.944, which was much lower than that of the best DT model (0.990). As for other five measurements, they are provided in Figure 3. It can be observed that each measurement was lower than that produced by the best DT model. This indicated the superiority of the best DT model. The employment of IFS method can help to build a more efficient model.

### Classification Rules

As described in the previous section, DT yielded the highest F1-measure on the COVID-19 methylation dataset when the top 50 features are used. Therefore, we applied DT to all samples using these 50 features to obtain six rules, which are provided in Table 1. Three rules each were related to COVID-19 and non-COVID-19. These rules clearly expressed the expression patterns of these features. These rules were described in detail in *Discussion Section*.

**TABLE 1:** Rules yielded by decision tree on top 50 features.

Index	Condition	Result
Rule0	cg03753191 ≤ 0.1398 cg15959262 > 0.5931 cg17439158 > 0.5681	Patient with COVID-19
Rule1	cg03753191 > 0.1398 cg17439158 ≤ 0.6170	Patient without COVID-19
Rule2	cg03753191 ≤ 0.1398 cg15959262 ≤ 0.5931 cg08399733 ≤ 0.9130	Patient without COVID-19
Rule3	cg03753191 > 0.1398 cg17439158 > 0.6170	Patient with COVID-19
Rule4	cg03753191 ≤ 0.1398 cg15959262 > 0.5931 cg17439158 ≤ 0.5681	Patient without COVID-19
Rule5	cg03753191 ≤ 0.1398 cg15959262 ≤ 0.5931 cg08399733 > 0.9130	Patient with COVID-19

## Discussion

Our research is dedicated to search for pathogenic clues of SARS-CoV-2 infection based on the methylation profiles of COVID-19 in confirmed and suspected patients. Epigenetic markers, such as histone modifications, DNA methylation, chromatin remodeling, and non-coding RNAs, can affect gene expression profiles and increase individual susceptibility to the virus. For example, DNA methylation is the basis for antigen presentation and host adaptive immune response in Middle East respiratory syndrome coronavirus infection (Menachery et al., 2018). Therefore, we aimed to explore how DNA methylation influences SARS-CoV-2 infection.

Meanwhile, our research proposed a novel and creative pattern with a high distinguishing degree in confirmed and suspected COVID-19 cases through MCFS. Although the real-time polymerase chain reaction test of sputum is the gold standard for the diagnosis of COVID-19, it takes a long time to confirm the diagnosis of patients because of the high level of false negatives. Therefore, researchers conducted various methods to better identify SARS-CoV-2 infection. (Hemdan et al., 2020) constructed a novel deep learning classifier to diagnose COVID-19 through X-ray images, which are cheaper, more convenient, and accessible compared with traditional chest X-ray and computed tomography. (Siddiqui et al., 2020) focused on the correlation of temperature with suspected, confirmed, and death cases by machine learning and found that temperature presents diverse trends in most cities and cannot be the decisive factor in different cases or situations. We focused on several top features and decision rules because they have a crucial impact on the classification and discussed them further through a wide literature publication to prove that our findings are reliable and convincing.

Epithelial stromal interaction 1 (*EPSTI1*, probeID: cg03753191) is an interferon (IFN)-responsive gene that was originally isolated from mixed cultured human breast cancer cells and fibroblasts (Nielsen et al., 2002). This gene is located on chromosome 13q13.3; is 104.2 kb in length; contains 11 exons; and is involved in tumor cell metastasis, epithelial–mesenchymal transition, chronic inflammation, tissue reconstruction, embryonic development, and other biological processes (De Neergaard et al., 2010). EPSTI1 plays an important role in the regulation of cell apoptosis. (Capdevila-Busquets et al., 2015) confirmed by *in vitro* experiments that EPSTI1 can inhibit breast cancer cell apoptosis by interacting with caspase 8. In addition, EPSTI1 has an antiviral effect against hepatitis C virus (HCV) by affecting the life cycle, viral replication, assembly, and release of HCV. (Meng et al., 2015) confirmed that EPSTI1 can promote the expression of protein kinase-R (PKR)-dependent genes, including *IFNβ*, *IFIT1*, *OAS1*, and *RNase L*, by activating the promoter of PKR to play an antiviral effect during the process of HCV infection. Without the involvement of IFN treatment, EPSTI1 overexpression effectively suppressed HCV replication, whereas the lack of EPSTI1 enhanced the viral activities. Current research discovered that EPSTI1 expression influences the performance of immune cells. (Kim et al., 2018) found that EPSTI1 expression is remarkably upregulated after macrophage activation with IFNγ and lipopolysaccharide. The proportion of M2-type macrophages is increased in the bone marrow-derived macrophage deficiency of EPSTI1. In EPSTI1 knockout mice, the number of M1 macrophage cells in the peritoneal cavity was significantly reduced. These findings demonstrate an important regulatory role of EPSTI1 in macrophage polarization. Therefore, EPSTI1 methylation may participate in the process of SARS-CoV-2 infection and affect inflammatory and immune function by regulating EPSTI1 expression.

NACAP1 (probeID: cg15959262) is a pseudogene of nascent polypeptide-associated complex-alpha (*NACA*). Phosphatase and tensin homolog (PTEN) pseudogene 1 (PTENP1) has been first revealed to contain microRNA response elements (MREs), which also exist in its corresponding protein-coding gene, *PTEN* (Poliseno et al., 2010). Increasing pseudogenes are found to have a similar phenomenon, that is, pseudogenes and their corresponding protein-coding genes function as competitive endogenous RNAs for binding to the same microRNAs (Lujambio and Lowe, 2012; Karreth et al., 2015). NACA encodes the *a* chain of nascent polypeptide-associated complex (NAC), which performs multiple functions, including protecting newborn peptides and regulating the translocation of new peptides into the endoplasmic reticulum and mitochondria (Rospert et al., 2002). The alpha chain of NAC alone acts as a transcriptional co-activator for developmental regulation (Yotov et al., 1998). Furthermore, NACA can regulate the conformation of Fas-associated death domain protein oligomer, which is an important mediator in the signal transduction pathway and can be activated by several members of the tumor necrosis factor (TNF) receptor family (Liguoro et al., 2003). NACA is related to neurodegenerative diseases. Patients with Alzheimer’s disease and Down’s syndrome have lower NACA expression levels in their brain cells (Kim et al., 2002). More importantly, the inhibition of NACA can induce the proliferation and differentiation of CD8^+^ T cells and enhance cytotoxicity. (Al-Shanti and Aldahoodi, 2006) used anti-sense technology to reduce the concentration of mRNA that translates NACA and found that CD8^+^ T cells will differentiate and activate to a higher degree in the presence of antisense oligonucleotide chains. Compared with the control group, the lethality of CD8^+^ T cells on target cells was enhanced.

SHROOM3 (probeID: cg17439158), a member of the Shroom family, encodes an actin-binding protein, which is important in epithelial cell shape and tissue morphogenesis (Haigo et al., 2003; Hildebrand, 2005). Shoom3 overexpression in epithelial cells induces rho kinase (Rock) recruitment and increases myosin 2 (Myo2) accumulation through phosphorylation and activation. The activation of the Rock/Myo2 signaling pathway leads to the local contraction of the actomyosin network on the top surface of the cell, which results in changes in cell morphology. Recent research has proved the indispensable role of SHROOM3 in glomerular filtration barrier integrity (Yeo et al., 2015). Forced Shroom3 expression in fawn-hooded hypertensive rat and endogenous shroom3 knockdown zebrafish improved kidney glomerular function. Moreover, multiple genome-wide association studies and *in vivo* experiments strongly demonstrated the correlation between SHROOM3 and congenital kidney disease (Khalili et al., 2016).

*C19ORF35* (probeID: cg08399733), also named *PEAK3*, is a member of the New Kinase Family 3 (NKF3) that can regulate cytoskeleton stability and cell motility by binding with an adaptor protein, CrkII (Lopez et al., 2019). C19ORF35 is associated with cancer progression. C19ORF35 overexpression has been detected in various cancers, including pancreatic, breast, and colon cancers (Wang et al., 2010; Kelber et al., 2012; Fujimura et al., 2014). C19orf35 methylation is related to early carcinogenesis. According to a DNA methylation sequencing study on 12 patients with early gastric cancers (EGCs), C19orf35 is remarkably hypomethylated in the diffuse type of EGC tissue compared with adjacent corresponding non-tumor mucosal tissue (Chong et al., 2014).

IFN-induced with helicase C domain 1 (*IFIH1*, probeID: cg21060789) and IFN-induced protein 44-like (*IFI44L*, probeID: cg13452062) are IFN-stimulated genes. IFIH1, also known as melanoma differentiation-associated gene-5, is a cytoplasmic RNA receptor protein composed of 1025 amino acids. IFIH1 recognizes double-stranded RNA with a length of more than 1 kb. It is an important member of the retinoic acid-inducible gene I (RIG-I)-like receptor family, which can activate type I IFN signaling pathway and participate in the pathogenesis of a variety of autoimmune diseases. IFIH1 and IFN-β interact to activate the body’s anti-tumor immune response. IFIH1 can promote type I IFN response and increase the secretion of TNF-α and IFN-β. The upregulation of IFIH1 expression may increase the effectiveness of IFN therapy (Pappas et al., 2009). IFN-β can also stimulate the upregulation of IFIH1 and RIG-I, mediate innate immune response, kill tumor cells with low neurotoxicity, and therefore inhibit tumor growth (Wu et al., 2017; Bufalieri et al., 2020). *IFI44L* is a paralog gene of *IFI44* and functions as a regulator of cell apoptosis, virus infection, and congenital immune response. The DNA methylation level of the IF144L promoter may be related to kidney damage in patients with systemic lupus erythematosus (SLE). (Zhao et al., 2016) found that the DNA methylation level of IFI44L promoter in patients with SLE was remarkably lower than that of the normal control group. In addition, the DNA methylation level of the IFI44L promoter in patients with SLE and renal involvement was also remarkably lower than that of patients with SLE without renal involvement. IFI44L participates in the antiviral process of IFN-mediated innate immune response and is a confirmed marker of early viral infection. IFN is the earliest discovered cytokine that can inhibit viral infection and replication and is activated in the early stage of viral infection (within a few minutes to a few hours) (Zaas et al., 2009). (Henrickson et al., 2018) pointed out that when influenza virus and respiratory syncytial virus infections occur, *IFI44L* acts as an IFN-stimulated factor regulatory gene, and its expression level increases. Therefore, the mRNA expression of *IFI44L* can be used as an early indicator of virus infection. According to (Kaforou et al., 2017), in 111 bacterial-infected children less than 60 days old, the detection sensitivity of IFI44L mRNA expression is 88.8% (95% CI, 80.3–94.5%), and the specific degree is 93.7% (95% CI, 87.4–97.4%). Therefore, aberrant IFI44L methylation may occur during SARS-CoV2 infection and lead to abnormal IFI44L expression.

*MX1* (probeID: cg26312951) belongs to human mycovirus resistance genes (*MX*) with biological functions, such as GTP binding and GTP enzyme activities. The two kinds of MX proteins, namely, MX1 and MX2, differ greatly in virus specificity and mechanism of action. MX1 has antiviral activity against a variety of RNA viruses and certain DNA viruses induced by type I and type II IFNs, including negative-strand RNA viruses and hepatitis B virus. MX1 is enriched in the IFN-γ and Toll-like receptor signaling pathways (Haller et al., 2015). A 2020 study detailed the expression of MX1 in 403 patients with COVID-19 and 50 patients without COVID-19 (Bizzotto et al., 2020). The expression of MX1, MX2, ACE2, and BSG/CD147 can cluster individuals with and without COVID-19 through principal component analysis, which indicated that the expression levels of MX1 and MX2 between patients with and without COVID-19 are remarkably different. MX1 can directly act on the ribonucleoprotein complex of the virus, so it has a wide range of antiviral activity. This feature has been proven to be suitable for RNA viruses and DNA viruses. (Verhelst et al., 2012) have reported that Mx1 interfering with functional viral ribonucleoprotein complex assembly led to inhibition of influenza virus and high MX1 expression can result in a better prognosis to influenza A (H1N1) pandemic in 2009. It is worth noting that GTPase activity is also positively correlated with its antiviral function. Different from MX1, the antiviral function of MX2 is limited to certain viruses, such as HIV. Although the expression of MX1 and MX2 in patients with COVID-19 were significantly higher than those in non-COVID-19 groups, MX1 shows a greater positive correlation with patients with COVID-19 and may be more specific than MX2 in response to SARS-CoV-2. Therefore, MX1 is a key responder to SARS-CoV-2 infection.

Collectively, the top identified discriminative feature genes and settled rules have a crucial role in virus infection and IFN-mediated immune response. This method demonstrates that our method is reliable and convincing. Our newly presented computational approach based on methylation profiles also provides a new perspective for exploring the mechanism of COVID-19. Furthermore, it is a new method that distinguishes confirmed and suspected COVID-19 cases and has applicable clinical value in the differential diagnosis of patients with confirmed and suspected COVID-19.

## Conclusion

The current study aimed to apply computational methods to extract the best biological features and decision rules from COVID-19 methylation profiles. This study has shown that the extracted optimal methylation site signatures and expression rules have been validated by previous work and are reliable and valid for distinguishing COVID-19. This study provides a new set of potential biomarkers/rules that can be used to differentiate patients with COVID-19 at the methylation level. These findings enhance our understanding of COVID-19 expression at the methylation level and could offer guidance for future studies on COVID-19.

## Data Availability

Publicly available datasets were analyzed in this study. This data can be found here: https://www.ncbi.nlm.nih.gov/geo/query/acc.cgi?acc = GSE174818.
